# On Cancer of the Mouth, Tongue, and Alimentary Tract

**Published:** 1886-12

**Authors:** 


*On Cancer of the Mouth, Tongue, and Alimentary Tract.* By F. B. JESSETT, F.R.C.S. Pp. 309. London: J. & A. CHURCHILL.

This is a plain, simple, unpretending work of average merit and evident good purpose. The author has had considerable opportunities of studying the diseases of which he writes, and with most of them he possesses a fair work-a-day experience. But Mr. Jessett's experience has evidently been more that of the passive recipient than of the active penetrating explorer. He has treated the diseases which came in his way up to his light and ability; but he has introduced no new thing, and he has not fully shown that he has made himself familiar with the original or even second-hand work of others. He is a safe guide for the general surgeon; he is in no sense a pioneer.

As a result of evident want of extensive reading, if not of want of discrimination, it would be easy to point to many examples of inaccurate balance in estimating the values both of theories and of practices. As examples of the latter, we may make selections from the admirable chapter on Cancer of the Intestinal Canal. On page 233 he says: "Mr. Treves and Mr. Bishop, of Manchester, have devised a form of clamp, which, while differing somewhat in make and shape, *are* [Italic ours] designated for the same purpose of clamping the gut above and below the part to be excised" . . . (in enterectomy). (By the way, Mr. Jessett's English is not that of Addison or Macaulay.) Now, we are far from saying that the clamps of Bishop and of Treves are not the best in existence; but while he is speaking in this general way of intestinal clamps, we think the name of Rydygier deserved mention for his instrument; and those of Makins (best of all, in our opinion) and Abbe were worthy of a passing notice.

On the next page (234) he says: "Two forms of sutures have been proposed and practised for uniting the divided ends of the intestines in these operations—the one by Czerny and Lembert, the other by Gussenbauer." . . . Two forms! one writer describes and depicts thirty-six! The Czerny-Lembert suture is undoubtedly a good one; but of the thirty-five other known sutures, Gussenbauer's is certainly not second best.

The otherwise admirable chapter on Cancer of the Tongue is somewhat marred by a want of precision in advice as to the selection of an operation, as well as of the mode of performing it. After reading his failure in an attempt to remove a tongue by Whitehead's method, we are left in doubt as to whether he has got the manual dexterity of a Whitehead; but we do not reject Whitehead's operation because it failed in Mr. Jessett's hands. "He would strongly recommend in all such cases as these that the lingual artery should be tied first." So would the reviewer, if the operator was not very skilful with his fingers; but this would not be Whitehead's method, and it would introduce an unnecessary element of danger in the double ligature of considerable vessels.

It would be possible to quote many examples of the faults we have found with the book. Its merits will be found on perusal. We believe that it is an honest record of such experience as the writer, with his lights and opportunities, has been able to gather; and that it gives a fair estimate of the value of so much of the work of others as he has been able to study. Not so much can be said for many medical works.

